# Promoting coordination in Norwegian health care^1^

**DOI:** 10.5334/ijic.581

**Published:** 2011-10-07

**Authors:** Tor Inge Romøren, Dag Olaf Torjesen, Brynjar Landmark

**Affiliations:** Centre for Care Research, Gjøvik University College, Teknologiveien 22, 2802 Gjøvik, Norway; Department of Political Science and Management, University of Agder, Service Box 422, NO-4604 Kristiansand, Norway; Centre for Care Research, Gjøvik University College, Teknologiveien 22, 2802 Gjøvik, Norway

**Keywords:** coordination, primary healthcare sector, secondary health care sector, reform plans, Norway, Denmark, Sweden

## Abstract

**Introduction:**

The Norwegian health care system is well organized within its two main sectors—primary health and long-term care on the one hand, and hospitals and specialist services on the other. However, the relation between them lacks mediating structures.

**Policy practice:**

Enhancing coordination between primary and secondary health care has been central in Norwegian health care policy in the last decade. In 2003 a committee was appointed to identify coordination problems and proposed a lot of practical and organisational recommendations. It relied on an approach challenging primary and secondary health care in shared geographical regions to take action. However, these proposals were not implemented. In 2008 a new Minister of Health and Care worked out plans under the key term “Coordination Reform”. These reform plans superseded and expanded the previous policy initiatives concerning cooperation, but represented also a shift in focus to a regulative and centralised strategy, including new health legislation, structural reforms and use of economic incentives that are now about to be implemented.

**Discussion:**

The article analyses the perspectives and proposals of the previous and the recent reform initiatives in Norway and discusses them in relation to integrated care measures implemented in Denmark and Sweden.

## Introduction

Promoting coordination between primary and secondary health care has been at the core of Norwegian health policy the last 10–15 years, like in many other countries.

Coordination here refers to mechanisms to integrate activities between health care organisations to facilitate appropriate service delivery. Two main initiatives and different strategies have been proposed during the last decade to foster better coordination, one in 2005 [1] and one in 2009 [2]. The first strategy soon was almost laid aside. The Government has now adopted a new health care legislation based on the second alternative and will implement administrative, structural and economic reforms from 2012 [3].

In this paper, we will describe and analyse the integrative health initiatives and proposals during the last years and discuss possible reasons for changes and postponements in Norwegian health policy. We will also discuss the Norwegian integrative health care strategies in relation to the policy previous implemented in Denmark and Sweden. These are the countries that are, at a general level, the most similar to Norway both politically, culturally and in terms of government system. Norwegian policy makers usually look to their nearest neighbours in order to learn or find solutions to organisational problems.

## The Norwegian health care system

Norway has a small and ageing population (4.5 million; 15% 65+) and a low population density (13 per square kilometer). Norwegian health and social care is still based on the classical Scandinavian Welfare model which combines financing and provision of universally accessible services mainly within the public sector. Total health spending in Norway accounted for 8.5% of GDP in 2008, compared with an average of 9.0% across OECD countries. Norway ranked the second highest among OECD countries in health spending per capita (512.000 NOK) [4].

Primary health care is provided under the responsibility of Norway’s 430 municipalities enacted in The Municipal Health Care Act from 1984. The municipalities are responsible for GPs, public health nurses, running nursing homes and home care. Nurses and doctors in preventive and long-term care services are usually employed in municipal health care. The services are financed through federal block grants, local taxation and out-of-pocket payments [5]. Municipalities spent almost one quarter of their total expenditures on health and care [6].

The municipalities are the lowest level of public administration and local democracy. Local government in Norway has strong standing and traditions since its founding legislation in 1837 [7]. However, many municipalities are small in terms of population. In 2008 as much as 50% of the Norwegian municipalities had <5000 inhabitants. On average a municipality has 10,000 inhabitants with a range from 250 to 500,000 people. The larger cities are subdivided into boroughs (city districts) covering services for about 30,000 inhabitants each. A municipality with 10,000 inhabitants will have about 10 GPs, 90 nursing home beds and 150 nurses, nurses aids and home helpers working in home care for elderly and disabled people [8].

Most general practitioners work as private contractors with the municipalities. Approximately 92% of the Norwegian GPs are private and are reimbursed ~30% per capita from the municipalities. The remaining 70% of their incomes come from co-payment from patients and reimbursement from Norwegian Labour and Welfare Services. Patients have a right to choose GP and 99% of the population is registered in a list system with on average 12–1500 patients per GP. GPs have a key role in the health systems as gatekeepers for the patients with regard to provision of health care services [9]. The Regular General Practitioner Scheme was introduced in 2001 and was in many ways inspired by the Danish organisation of GPs [10]. One consequence was that the patients’ rights to choose GP, at least formally became more restricted [11]. However, patients were still free to choose their physician outside their own municipality or district.

The hospital sector of Norway is responsible for the specialist health care service and has been run and owned by national health authorities since 2002 when a major hospital reform took place. The reform contained two major changes. Firstly, the ownership of all public hospitals was transferred from the 19 counties to the state. Secondly, the government decided to set up five (later four) Regional Health Enterprises to manage and run Health Enterprises (HE) [12]. During the last decade the sector has been restructured and previous single hospitals have merged into larger enterprises. Fifty-five hospitals (1999) have been reduced to 21 health enterprises (2011) [10, 13].

The health enterprise boards are responsible for organising a complete set of acute somatic and psychiatric specialist services to the population in the area. Acute somatic hospitals vary from small local institutions with basic medical and surgical services to larger hospitals with a wider spectrum of specialist services and hospitals affiliated with a university offering medical education (one teaching hospital in each region). Except for a few institutions with advanced rehabilitation services, long-term care does not exist within the hospital sector in Norway. It is, as mentioned above, integrated in primary health care.

The hospital sector is financed through government grants. The health enterprises are reimbursed with ~40% depending on their DRG-based activity. Sixty percent is block grants [14]. The patients’ out-of-pocket payments applies only for ambulatory services. Private health insurance plays a marginal role in funding of Norwegian health services, estimated at 1.8–2.3% [15].

Although patients in principle are free to choose whatever hospital they want, most GPs and patients will choose one within their immediate geographical region. Therefore, there is a lot of interchange of patients and tasks between each hospital and the primary health and long-term care in the municipalities in the surrounding area. Each hospital cooperates with several different municipal health services, with a range from two to more than 30.

This health care system is fairly systematically organised within each sector—primary health and long-term care on the one hand, and acute somatic and psychiatric hospitals and specialist services on the other. But the relation and interaction between the two lacks mediating structures. This weakness is further aggravated by the fact that each sector belongs to separate levels of public administration: local and national. The two sectors have different systems of funding and different administrative, political and professional cultures. The specialist health care sector has high competence, and can be considered to be highly medical and diagnostic intensive. Municipality health services are characterized by lower skills, where as much as 29% of the labor force is performed by personnel without appropriate formal health professional education, mostly in long-term care. In both hospitals and municipal health services reforms and developmental work have been triggered in each sector by its own culture and organisational rationality [9]. Such a construction may obviously contain barriers to good vertical inter-organisational coordination. In many ways these differences have ridden the Norwegian health care system and have led to coordination problems that have never been resolved since the municipality health care act came in 1984 [16].

Not surprisingly, national health authorities have been looking for strategies to reduce negative effects of this two-level model.

## Integration policy

Over the years numerous local initiatives and projects regarding coordination have been launched spontaneously both by hospital leaders, primary health care authorities and professionals [17]. Projects have often been targeted at special patient groups, like cancer patients, elderly or psychiatric patients. Such local projects have usually been implemented for idealistic or professional reasons, without economical or formal support from any health authority. A good example is the employment of practice consultants and practice coordinators (PCs) in the health enterprises, organised through the Norwegian Medical Association. GPs are employed in hospitals for about 10 hours a month. Their role is to contribute to better cooperation and patient-flows and identify areas of improvement between the primary services and the specialist health care [18].

Cooperation efforts have also been set up on a national level. In 2001 a mandatory individual care plan was introduced. This arrangement gave patients with complex or chronic health problems the right to receive managed and coordinated care and to be involved in planning their health and social services. Despite the arrangement being obligatory by law, the outcomes have showed a low number of care plans and lack of responsibility among professionals at the operative level. National objectives have not been achieved in this area [19].

The first general national policy initiative for better integration in health care was taken in 2003 when a committee (“The Wisløff committee”) was appointed by the government and asked to 1) identify coordination problems in the Norwegian health and long-term care services, and 2) propose practical solutions to strengthen coordination within the total service system. Research in the area is still sparse in Norway, so the committee’s report [1] relied heavily on practical experience and interesting cases. On this background, the report pointed to six different patient groups particularly vulnerable to coordination problems: cancer patients, elderly with high comorbidity, the terminally ill, patients with chronic disease, psychiatric patients and drug abusers.

The proposals in the report targeted the operational level of integration. The report considered the implementation of many of its proposals to be up to regional and local health care agencies. However, at one point the report pointed at a measure on national, administrative level: writing agreements between primary and specialist health care on routines for hospital admissions and discharges.

The committee proposed a system of agreements between hospitals and the nearby municipalities throughout the whole country. These agreements aimed at reducing unnecessary admissions, reducing waiting time before hospital discharge, and to make transitions from hospital to home as efficient and safe as possible for the patient.

The committee had a sharp eye for the power game between primary and secondary health care, with the latter as the strongest part. It argued for equalization as an important prerequisite for developing sound coordination. The report argued against main economic or organisational reforms as an effective means to foster coordination, but opened up for trials in this area to gain experience before any major structural reform was to be launched.

Of the many recommendations put forward by the Wisløff-committee, The Ministry of Health and Care (with a new minister from 2005 to 2008) primarily invested political awareness and administrative capacity only in developing the formal agreements system on a national scale. This process was completed in 2008; all the hospitals have formal agreements with their surrounding municipalities [20].

## The new reform initiative

Entering office in The Ministry of Health and Care during the fall of 2008, another minister defined coordination in health and long-term care as his key interest and primary political priority. Very soon he was engaged in working out new plans under the key term “Coordination Reform”. In June 2009 Report No. 47 (2008–2009), “The Coordination Reform” was passed to the Norwegian Parliament (Storting) [2].

This report represents a shift in perspective away from the operational to the administrative level and appeals for the need for economic or organisational reforms. It pointed at the consequences of demographic changes for health care utilization and proposed major structural reforms to reduce the demand for hospital services. This perspective is well in tune with WHO’s arguments for the need for integrated care [21]. In this proposal, vertical integration in health care became a means to foster cost containment and not primarily to monitor patient careers safely and effectively. Again, the problem identification and the proposed solutions were not well documented with literature from the research community.

Key features of the “Coordination reform” are two well-known strategies put forward in many health systems: 1) more patients should be taken care of in primary health and long-term care instead of being referred to hospital treatment; and 2) discharge from acute hospitals should take place earlier. The report proposed strong economic incentives to underpin these strategies: funding the establishment of pre-hospital low threshold wards in primary health care and introducing a co-funding from primary health care to hospitals. This co-funding includes municipal co-payment of general hospital admissions and a penalty fee for not immediately receiving patients ready for discharge from the same institutions in need of rehabilitation or long-term care.

In contrast to the Wisløff committee report this new Coordination reform plan targets the administrative level and relies heavily on economic incentives. Instead of trying out organisational and economical means on a limited scale, the “Coordination Reform” attacks the problems straight on with a radical and ambitious reform program. The main tools for achieving better coordination and integration are economic incentives, legal means and restructuring tasks and responsibilities between the specialist and primary health care sector.

The Norwegian Parliament (Storting) gave response to the report during the spring of 2010 [22]. It expressed uncertainty about the economic measures and the time frame of the reform plans. Afterwards, the Ministry of Health spread out elements of the “Coordination Reform” in different legislative documents and by a hearing. A new health and care plan and legislation was decided to be implemented by the Government in April 2011. In June 2011 the reform-legislation passed the Storting just before adjournment for the summer [15]. All the three main elements from the proposals back to 2009 were approved. The penalty fee and co-funding will be introduced from 2012. Co-funding is limited to 20% of hospital costs for all patients with medical diagnoses. Money for these expenses will be transferred from hospital to municipal budgets. Low threshold wards in the communities must be ready to be set into operation in 2016, funded by money saved by reducing the stream of patients with medical diagnosis from communities to hospitals [23].

## Denmark and Sweden

The two other Scandinavian countries have health care services based on the same principles and with much of the same structure as Norway: universal access, a dominant public financing and provision, and different levels of public administration in charge of primary and secondary health care. In addition, questions of coordination and integration have been central to health policy initiatives across all three countries during the last decade. Many of the policy initiatives and formal arrangements are similar [24]. However, there are also differences. It is interesting to note the correspondence between the two alternative Norwegian strategies and integrative health policy measures in Denmark and Sweden. In Denmark most initiatives are induced centrally and they primarily target the administrative level, partly based on formal legislative revisions and economic incentives [24]. This central approach is in tune with the strategies of the Norwegian “Coordination reform”.

In Sweden, local solutions are more prominent, and policy initiatives typically more directed at the operational level [24]. This is well in tune with the views and proposals of the Wisløff committee. In Sweden and Denmark the digitalisation of health care is obviously more advanced than in Norway [24]. In Norway, an array of technical, political and administrative obstacles have led to major postponements in the implementations of the health care information technologies. However, in the wake of the reform the Government has announced that a national cross-sector eHealth integrative committee will be appointed soon. In many ways this is an initiative parallel to the strategy for digitalisation of the Danish healthcare in 2007 [24].

Agreements between hospitals and municipal authorities are a common feature both in Denmark and Norway. This practice is lacking in Sweden which on the other hand has developed a comprehensive system of “chains of care” [25, 26]. These are patient pathway descriptions regulating activities within healthcare involving several providers and aim at creating organisational links. In addition, hospital doctors are required by law to inform the primary health care system about hospitalization and rehabilitation plans in due time before patient discharge [27]. Again, this shows characteristics of the Swedish approach: operationally orientated and relying on local initiatives. Economic incentives like municipal co-payment for hospital admissions (lacking in Sweden), have been imported from Danish health policy into the Norwegian “Coordination reform”. In the same way a “Local care” concept is imported from Sweden, implying a more flexible hospital system, e.g. low threshold pre-hospital wards in primary health care [28]. In the Norwegian reform proposal extensive legislation is used to “secure” cooperation between service providers, i.e. that municipalities and hospitals are obliged to appoint a coordinator for patients who need long-term and coordinated services [3].

All in all, one may find common elements in healthcare integration policy across all the Scandinavian countries. However, Sweden and Denmark stand out with two distinct approaches. During the recent years Norwegian health policy has been tottering between the two.

## Discussion

Why has the health policy of integration in Norway changed from a mainly local and task-oriented strategy to a mainly administrative, economically oriented approach? Since the appointment of the Wisløff committee in 2003, there have been five different ministers in the Norwegian Ministry of Health and Care. This may indicate that health policy at a national level is a difficult and person-consuming activity. However, most changes have been due to elections and mid-term reshuffling. The three last ministers were all from the same party (Labour). Therefore, the shift in strategy in 2008 had little to do with major political changes. More likely, different opinions about effective and reliable administrative tools in health care, increasing health expenditures and policy learning from WHO and Denmark may explain the changes.

In contrast to many other countries, the economy of the public sector in Norway so far has not been seriously affected by the international financial crisis. Major cutbacks do not acutely threaten health care like in other countries. However, as public expenditures on health increase, so does the probability of policy change [29]. Norway, like other countries, seems worried about increasing health expenditures in the long run. The health enterprise reform in 2002 did not give the expected economic outcome and expenditures in specialist health care have doubled since. Bjarne Håkon Hansen, the minister of Health and Care who announced the Norwegian Cooperation reform stated that: *“Norway spends the most money in the world on health. Expenditure on hospitals has doubled since 2002, but we do not get enough value for money for all this spending. If we continue like this, the health care system will break apart”* [30, p.1].

Will the coordination reform reach its goals?

In line with the numerous grass root initiatives put in place before any national integration policy arose, low-threshold wards in primary health care already exist in some municipalities, typically in medium sized cities [31]. This type of health care was originally established for rehabilitating elderly patients after acute hospital treatment. The initiative has been considered to be successful both in terms of improved quality and costs [32]. The Government now will implement such units all over the country planning to have this new health infrastructure operative before 2016. Primary health care in the municipalities is encouraged to use these wards as an alternative to hospital treatment, not only for rehabilitation purposes. This goal displacement will most likely lead to stronger rationing of hospital resources for elderly and chronically, ill patients. Whether this will be an advantage in terms of treatment quality or costs is to our opinion, an open question at the moment.

The reform could also represent a heavy financial burden and risk for the many small Norwegian municipalities that already are in a stressful economic situation. The capacity in the nursing home and home care sector is under pressure [33], and there is already little slack and inadequate medical expertise in many municipalities [34]. In the end, this stressful situation could also result in lower service quality.

The effect of a penalty fee for not immediately receiving patients ready for discharge from somatic hospitals and in need of rehabilitation or long-term care will probably be weak. A recent study shows that this patient group use about 4% of the in-patient bed-days in somatic hospitals today. This share has been decreasing over the last years [35]. A penalty fee may reduce this phenomenon further, and probably prevent it from increasing again. But as a means for increasing hospital effectiveness the intervention will be a modest contribution, at least in the short run.

Solutions taken from Danish and Swedish health policy have now been adopted in the Norwegian integrative health policy. However, the Norwegian reformers seem not to have learned much about their Scandinavian neighbours. All the three countries have introduced different forms of quasi-market and increased the use of economic incentives. Experiences and learning from Sweden tell us that, despite new initiatives for better chains of care, a parallel policy with increasing economism has an adverse and fragmenting effect on development on integrated care [25].

The lack of learning also seems to be the case when Norway adapts to the Danish model of incentives and municipality co-payment for specialist health care. The Danish experiences tell us that this policy may lead to fragmentation and competition rather than cooperation between sectors in health care. The co-payment arrangement in many cases is considered to be ineffective [36].

In the new reform proposal the Norwegian government relies on formal legislation for improving coordination. But formal legal framework is no warranty in this field. Experiences from previous legislation both in Norway and Denmark tell that a change of legislation did not necessarily provide better cooperation at the operational level [19, 24].

Why not integrate Norwegian primary and secondary health care in one administrative body?

Surprisingly this has not been proposed as an option so far in Norwegian health policy. The fundamental coordination-problems between two different political, administrative and professional cultures and sectors will still be present if the new health legislation is being implemented [16]. The reason for why one administrative body has not been discussed in the new reform initiative may be that decentralization is an important value in the Norwegian political culture. Moving the responsibility for primary health and long-term care from local to central political and administrative authorities therefore represents a radical shift in government, which most likely will foster strong resistance [7]. One may also argue that full administrative and economic integration of primary and secondary health care does not necessarily solve problems of coordination on the operative, patient-oriented level [37].

Historically, the Norwegian local communities have been the “avantgarde” in taking new initiatives in health care. This has been called “welfare localism” [38]. However, due to the fact that many Norwegian municipalities are very small and lack sufficient resources and competence, inter-municipal collaboration can be an alternative to structural reforms when compulsive merging of municipalities probably will lead to heavy political resistance in rural communities. Inter-municipality cooperation is relatively easy to customize and gives the possibility for more efficient and competent service delivery. At the same time it safeguards local autonomy [39]. With respect to health care, there are probably scale benefits at a certain population level. The government has recommended that the optimal sufficient size for service delivery in primary health care is an average population of 20,000 [2].

## Conclusion

Integration policy has been high up on the agenda for Norwegian health authorities during the last decade and two major national policy initiatives concerning cooperation have been promoted: the Wisløff committee [1] and the cooperation reform proposal [2]. There are similarities and continuation between them, but also a change in focus where the latter take stronger economic, legal and organisational restructuring measures into use. The cooperation reform with its administrative and regular approach represents a change in strategy from the mainly voluntary and task oriented Wisløff-committee recommendations. Both approaches have borrowed elements from the same policy areas implemented in Sweden and Denmark. The final law proposals, however, have most in common with recent Danish integration policies.

The main shift in focus and changes in strategy from an operative to an administrative strategy, seems to be explained by increasing health expenditures, that previous reform has not delivered its economic prospects, and policy learning from WHO and Denmark.

Although significant learning takes place when it comes to recipes for better coordination in health care, it does not appear that the Norwegian reformers have learned extensively about the effects of them discussed in the scientific literature. This may lead to problems with goal attainment for the coordination reform in Norway and trigger off possible negative side-effects as well.

## Reviewers

**Bengt Ahgren**, PhD, MPolSc, Associate Professor, Nordic School of Public Health, P.O. Box 12133, SE-402 42 Göteborg, Sweden

**Rasmus Antoft**, PhD, Associate Professor, Head of Department of Sociology and Social Work, University of Aalborg, Denmark

**Corinne Grenier**, Professor, Scientific Director of the Social and Healthcare Cluster, Euromed Management, Researcher affiliated with the CERGAM Laboratory (IMPGT Team), University of Aix-Marseille III, France
